# Impacts of Atmospheric and Anthropogenic Factors on Microbiological Pollution of the Recreational Coastal Beaches Neighboring Shipping Ports

**DOI:** 10.3390/ijerph19148552

**Published:** 2022-07-13

**Authors:** Romina Kraus, Vanja Baljak, Darija Vukić Lušić, Lado Kranjčević, Arijana Cenov, Marin Glad, Vesna Kauzlarić, Dražen Lušić, Luka Grbčić, Marta Alvir, Marijana Pećarević, Slaven Jozić

**Affiliations:** 1 Center for Marine Research, Ruđer Bošković Institute, Giordano Paliaga 5, 52210 Rovinj, Croatia; kraus@cim.irb.hr; 2 Faculty of Medicine, University of Rijeka, Braće Branchetta 20, 51000 Rijeka, Croatia; vanjabaljak@yahoo.com; 3 Department of Environmental Health, Teaching Institute of Public Health of Primorje-Gorski Kotar County, Krešimirova 52a, 51000 Rijeka, Croatia; arijana.cenov@zzjzpgz.hr (A.C.); marin.glad@zzjzpgz.hr (M.G.); 4 Department of Environmental Health, Faculty of Medicine, University of Rijeka, Braće Branchetta 20, 51000 Rijeka, Croatia; drazen.lusic@medri.uniri.hr; 5 Center for Advanced Computing and Modelling, University of Rijeka, Radmile Matejčić 2, 51000 Rijeka, Croatia; lgrbcic@riteh.hr; 6 Department of Fluid Mechanics and Computational Engineering, Faculty of Engineering, University of Rijeka, Vukovarska 58, 51000 Rijeka, Croatia; malvir@riteh.hr; 7 Department of Environmental Health, Institute of Public Health of Istrian County, 52100 Pula, Croatia; vesna.kauzlaric@zzjziz.hr; 8 Department of Basic Medical Sciences, Faculty of Health Studies, University of Rijeka, Viktora Cara Emina 5, 51000 Rijeka, Croatia; 9 Department of Applied Ecology, University of Dubrovnik, Ćira Carića 4, 20000 Dubrovnik, Croatia; marijana.pecarevic@unidu.hr; 10 Institute of Oceanography and Fisheries, Šetalište I. Meštrovića 63, 21000 Split, Croatia; sjozic@izor.hr

**Keywords:** Adriatic Sea, microbial pollution, fecal bacteria, shipping ports, coastal bathing water

## Abstract

A comparative study of the two northeastern ports of the Adriatic Sea indicated that the port of Rijeka is microbiologically more loaded than the port of Pula and posing a greater threat to other ports through a potential transfer of pathogens by ballast water. Fecal indicator bacteria, *Escherichia coli* and intestinal enterococci, were investigated seasonally in 2014–2015 in the ports and during the bathing season monitoring in the two bays where ports are located in 2009–2020. In addition, the indicators and pathogens related to human health were determined in the ports’ seawater and sediment. The determined factors contributing to microbiological pollution were higher number of tourists and locals, potential wastewater and ballast water discharge and enclosed port configuration, with high solar radiation and low precipitation reducing the negative effects. Our research points to the necessity of including *Clostridium perfringens* in monitoring beach sand during the bathing seasons and a wider list of pathogens in port monitoring due to a potential transfer by shipping ballast water.

## 1. Introduction

The decline in seawater quality in coastal areas is a major challenge, given the ever-increasing pressures caused by human activities. According to current estimations, over 40% of the world’s population lives within 100 km of a coastline, with a tendency of constant growth [1]. Urbanization, industry, tourism, aquaculture and shipping are just some of the anthropogenic activities affecting this sensitive system. The impact is particularly pronounced in larger seaport cities, where numerous port activities impose an additional source of a diverse range of pollutants on the surrounding areas, which are often important recreational and tourist areas. 

The current EU Bathing Water Directive (EU BWD) [2] defines bathing water quality categories based on two general fecal indicator bacteria (FIB), *Escherichia coli* (*E. coli*) and intestinal enterococci, the most commonly used indicators of fecal pollution and possible presence of pathogens. The determination of pathogens in a routine assessment of microbial contamination is technically and financially infeasible, given the large number of microorganisms that should be determined and the underdeveloped methods that are often time consuming and laborious. However, for a more comprehensive assessment of microbial contamination, many authors suggest occasionally including additional indicators and pathogens, such as *Clostridium perfringens* (*C. perfringens*), *Staphylococcus aureus* (*S. aureus*), *Pseudomonas aeruginosa* (*P. aeruginosa*), *Salmonella* spp., *Shigella* spp. and *Vibrio* spp. [3,4,5,6,7]. Microbial contamination prediction methods prove to be a feasible tool in water quality control [8,9]. Additionally, during the assessment of microbiological pollution sources, the sediment and the beach sand are only sporadically examined, although the data indicate that this habitat could serve as an important reservoir of microorganisms [10,11,12,13]. Moreover, the WHO’s recent guidelines on recreational water quality recommend including the microbiological quality control of beach sand in regular monitoring [14].

Among the many sources of coastal microbial pollution, shipping ballast water (BW) has been identified as an emerging issue [15,16]. Ballast water is water pumped as ballast into the tanks of steel-hulled vessels to stabilize the vessel at sea. This practice reduces stress on the hull, provides transverse stability, improves the propulsion and maneuverability and compensates for weight changes at different levels of cargo loading and due to fuel and water consumption. Aiming to prevent the transport of invasive aquatic species associated with global marine traffic, the International Maritime Organization [17] has adopted the International Convention for the Control and Management of Ships’ Ballast Water and Sediments. The Convention also specifies the maximum allowed amounts of discharged viable organisms and indicator microbes during BW discharge, *E. coli*, intestinal enterococci and *Vibrio cholerae* (*V. cholerae*), as part of the D-2 standard. The studies on microbial contamination of seaports and their potential to pollute adjacent coastal areas are rather limited [11,18,19,20,21,22,23], and there is a need for further research. 

The objective of this study was to provide a more comprehensive assessment of microbial contamination of seawater and sediments in two ports in the northeastern Adriatic Sea (the northernmost part of the Mediterranean Sea) in terms of the impact of the ports on the surrounding recreational area and the potential impact of contaminated ballast water transfer to other areas. A numerical model of the ports’ impact on water quality was also built. In addition, the long-term microbiological quality of seawater in the coastal area surrounding the ports and in the wider coastal area was assessed according to the EU BWD [2], and the possible atmospheric and anthropological influences on the studied areas were numerically examined.

## 2. Materials and Methods

### 2.1. Study Areas

**Figure 1:**
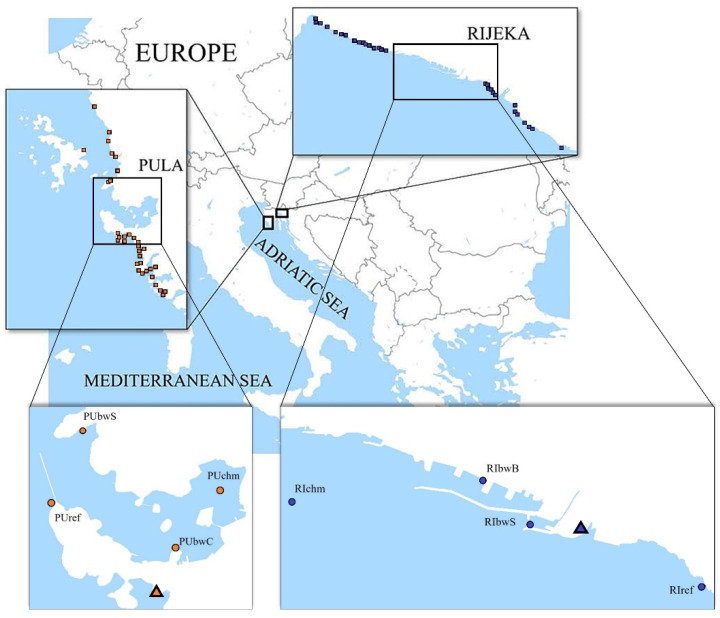
Maps showing locations of sampling sites (circle) and wastewater treatment plants (triangle) in the two investigated areas: the Istria County with the port of Pula and the Primorje-Gorski Kotar County with the port of Rijeka.

Two areas located in the northeastern Adriatic Sea, the Pula Bay and the Kvarner Bay (also known as the Rijeka Bay), which host shipping ports of different traffic intensity, were selected for the study (Figure 1). Approx. 8000 m^3^ of BW was discharged monthly in the port of Pula and 50,000 m^3^ in Rijeka in the 2012 to 2015 period [24]. 

The port of Pula occupies nearly the entire Pula Bay. Located in the deepest gulf on the Croatian coast (average depth 25 m, max 35 m), it is one of the largest (~8 km^2^) and best protected ports/bays in Croatia. Inside the port of Pula, there are no large man-made or natural barriers. However, the natural, rather narrow entrance to the bay was further narrowed by the construction of a 1210 m long pier, leaving a passage width of 400 m, restricting the exchange of waters with the open sea. The port is therefore well protected from adverse conditions from the open sea and the winds. The configuration also prevents the formation of high waves. The highest waves result from the strong NE and N winds (Bura and Tramontana), up to a height of 0.25–0.30 m [25]. The Valkane wastewater treatment plant in Gortanova Bay, 1.5 km away in direct line from the port of Pula, has a capacity of up to 39,000 m^3^/day of mechanical pre-treatment and a 1.356 m long submarine outlet with a diffuser.

The port of Rijeka, the biggest port in Croatia, consists of several terminals located all over the Kvarner Bay, which encompasses over 115 km of the coastline. The Bay spreads over an area of ~450 km^2^, with an average depth of 60 m (max 67 m), and is of a closed type, without any natural barriers to marine traffic. The strongest and most frequent wind in the area of Rijeka Bay is Bura, a NE wind, especially in the cold period of the year (winter and early spring), although with half the frequency in the summer also. Blowing in short bursts, Bura often reaches the hourly averages of above 20 m/s, even up to 35 m/s. The second important factor regarding the maximal speed and frequency is the ESE/S wind (Jugo), especially in the winter months, blowing for 2–3 days, sometimes up to a week. When attaining significant strength, Jugo induces a very rough sea. Another significant and strong wind is the SW wind (Lebić), occasionally attaining storm strength [26]. A wastewater treatment plant with mechanical pre-treatment and a 548 m long submarine outlet with a diffuser and a capacity up to 43,200 m^3^/day are located in the very center of the city, 700 m away in direct line from the port of Rijeka.

In the Adriatic, the sea tides are of a mixed type and most pronounced in the northern part. Additionally, the sea level is influenced by atmospheric processes. Interestingly, in the northern Adriatic, the sea level occasionally rises up to 1 m during strong S/SE winds with rather low barometric pressure, while during the NE wind Bura episodes with high barometric pressure, the sea level can decrease up to 1.2 m. Moreover, in the northern Adriatic bays, free oscillations (seiches) occur, which can induce sea level variations of up to 50 cm.

In each port, four sampling sites were selected (Figure 1). Two were commercial shipping facility locations and therefore directly influenced by BW. In the port of Pula, the two sampling sites directly influenced by BW were a cement terminal (PUbwC) and a stone terminal (PUbwS), both open coastal sites. In the port of Rijeka, the two sampling sites directly influenced by BW were a grain terminal (RIbwB) in the basin of Rijeka and a general cargo and timber terminal (RIbwS) in the basin of Sušak, each enclosed by a breakwater measuring 1754 m and 420 m, respectively. In each port, a third site was chosen at a location indirectly influenced by BW inside the Pula Bay (PUchm) and near the entrance to the port basin of Rijeka (RIchm). A fourth site was selected outside the port, as a control site, assumed without or under limited BW influence (PUref, RIref).

Additionally, the investigation was performed at 488 nearby sites (Figure 1), recreational coastal waters spread along the coastline in a wider area of the Istria County (IC) and Primorje-Gorski Kotar County (PGKC), encompassing the Pula Bay and the Kvarner Bay, respectively. Each area was divided into the recreational coastal area surrounding the port (surrounding area) and the remaining wider coastal area of the county (wider area). In total, 270 sites were monitored in PGKC (wider area: 1065 km of coastline; surrounding area: 15 km of coastline) and 218 in IC (wider area: 1056 km of coastline; surrounding area: 13 km of coastline). There were 29 sites (16 west, 13 east) in the surrounding area of the port of Rijeka and 31 sites (7 west, 24 east) in the surrounding area of the port of Pula (Figure 1). In total, 53,679 samples were analyzed: 29,956 in PGKC and 23,723 in IC.

### 2.2. Sample Collection

#### 2.2.1. Shipping Ports

The sampling in ports was conducted seasonally, in the autumn (September and November 2014 and November 2015), winter (December 2014 and February 2015), spring (April–May 2015) and summer (July 2015). Seawater samples were collected manually from a depth of approximately 30 cm using a 2 L sterile glass bottle. Scuba divers collected the sediment samples from shallow sites with 1 L plastic containers. A Van Veen grab sampler was used for sampling at deeper sites. From the upper sediment layer in the grab sampler, subsamples were collected manually. Samples were stored at 4 °C until the transfer to the laboratory for immediate processing. 

#### 2.2.2. Coastal Recreational Waters

Seawater samples were collected fortnightly during the bathing season (mid-May to late September), a total of 10 samples per location per season. Sampling was carried out at official sites, in the morning, and according to a pre-established monitoring calendar. Samples were taken from approximately 30 cm depth using a hand sampler and sterile 500 mL glass bottles. The samples were stored in transport refrigerators at 4 ± 3 °C and analyzed immediately upon arrival in the laboratory.

### 2.3. Microbiological Analysis 

Microbiological parameters in the seawater and the sediment were determined using culture-based methods. The following bacteria were examined: *E. coli*, intestinal enterococci, *Vibrio alginolyticus* (*V. alginolyticus*), *Vibrio cholerae* (*V. cholerae*) non-O1/non-O139, *Vibrio fluvialis* (*V. fluvialis*), *Vibrio metschnikovii* (*V. metschnikovii*), *Vibrio parahaemolyticus* (*V. parahaemolyticus*), *P. aeruginosa*, *S. aureus*, *C. perfringens*, *Salmonella* spp., *Shigella* spp., *Aeromonas hydrophilla* (*A. hydrophilla*) and *Aeromonas sobria* (*A. sobria*). For all parameters, 100 mL of the water sample or an appropriate dilution (1:10, 1:100) was used, except for *Salmonella* spp. and *Shigella* spp. for which 1000 mL of the sample was filtered. Sterile gridded mixed cellulose ester membranes, 47 mm diameter, pore size 0.45 μm (GN-6 Metricel^®^, Pall Corporation, Ann Arbor, MI, USA), were used, except for *S. aureus*, for which sterile 0.2 μm pore size mixed cellulose ester membranes were used (Whatman^®^, Maidstone, UK).

To prepare the sediment for analyses, 50 g was placed into sterile stomacher bags and phosphate-buffered saline (PBS, ratio 1:10) with 0.5% Tween 80 added. The samples were periodically hand homogenized for 30 min. After mixing and settling, 25 mL of the eluent was decanted and processed for all specific parameters, except for *Vibrio* spp., *Salmonella* spp. and *Shigella* spp., where 100 mL was used. The sediment eluents were processed by the membrane filtration technique, using the same methods and membranes as for the seawater samples.

The ISO 9308-1: 2000 method was used for *E. coli* determination. The membrane was incubated on a two-layer Tryptone Soy Agar/Tryptone Bile Agar (TSA/TBA) medium, 4–5 h at 36 ± 2 °C followed by 19–20 h at 44.0 ± 0.5 °C. The confirmation test was based on the indole production where the membrane with grown colonies was placed on an adsorbent pad saturated with Indol rapid reagent and exposed to UV radiation for about 20 min. All red colonies were considered as *E. coli*. Since 2015, a revised version of this standard has been applied (ISO 9308-1:2014) based on a positive β-D-galactosidase and β-D-glucuronidase reaction after incubation at 36 ± 2 °C for 21 ± 3 h on Chromogenic Coliform Agar (CCA) agar. The characteristic *E. coli* colonies appeared as dark-blue to violet color.

The intestinal enterococci were determined using the ISO 7899-1:1998 method. The membrane was incubated on selective SBA (Slanetz and Bartley Agar) medium at 36 ± 2 °C for 44 ± 4 h. A confirmatory test was performed by transferring the membrane with grown colonies onto Bile-Aesculine Agar (BAA) medium (at 44.0 ± 0.5 °C for 2 h), where aesculin hydrolysis resulted in the formation of a black halo around the intestinal enterococci colonies.

*P. aeruginosa* was determined in accordance with ISO 16266:2008. The membrane was incubated at 36 ± 2 °C for 44 ± 4 h on CN agar (Pseudomonas Agar Base/CN-agar). All green/blue colonies were counted as *P. aeruginosa*. All the colonies fluorescent under Wood’s lamp and reddish-brown colonies that did not fluoresce were considered as presumptive. Presumptive colonies were confirmed by testing the organism’s ability to utilize acetamide using Acetamide Broth, an oxidase test and King’s B medium that promotes the production of fluorescent pigment at 36 ± 2 °C for up to 5 days.

The enumeration of *S. aureus* was performed in accordance with the APHA St. Methods 9213B, 22nd Ed using the BP agar (Baird Parker agar medium + Egg Yolk Teluritte Emulsion). After incubation at 36 ± 2 °C for 44 ± 4 h, the typical colonies of *S. aureus* were black to gray, shiny, convex (1–2.5 mm in diameter), surrounded by a clear halo zone (visible below the filter). Gram staining and coagulase tests were used as confirmatory tests. The presumptive colony was transferred to a tube with a Brain–Heart Infusion broth and incubated at 36 ± 2 °C for 22 ± 2 h. Afterward, the rabbit plasma with bacteria was inoculated at 36 ± 2 °C for 22 ± 2 h. The formation of a clot indicated coagulase production.

*C. perfringens* determination was performed in accordance with the EU Directive (98/83/EC) [27]. After filtration, the membrane was placed on an m-CP Agar Base (Membrane *Clostridium perfringens* Agar Base) and incubated anaerobically in gas jars at 44 ± 1 °C for 21 ± 3 h. The straw-yellow colonies on the m-CP Agar Base, which turn pink or red after 20–30 s exposure to ammonia vapors due to the expression of acid phosphatase, were regarded as *C. perfringens*.

The isolation of *Vibrio* spp. (*V. alginolyticus*, *V. cholerae* non-O1/non-O139, *V. fluvialis*, *V. metschnikovii*, *V. parahaemolyticus*) was performed in accordance with the standardized method for food and animal feed ISO/TS 21872-1:2007/Cor 1:2008, modified for the water matrix. A 100 mL water sample was filtered (47 mm diameter, pore size 0.45 μm, GN-6 Metricel^®^, Pall Corporation, USA), and the membrane was incubated on TCBS medium (Thiosulfate citrate bile and sucrose agar) at 37 °C for 24 h. Another 100 mL of the sample was added to a bottle with 900 mL of Alkaline Saline Peptone Water (ASPW) enrichment media and incubated at 41.5 °C for 5–7 h. Afterward, a 1 mL aliquot of the suspension was transferred to a tube with 9 mL ASPW and incubated at 41.5 °C for 17–19 h. The cultures obtained in the ASPW were then streaked onto a TCBS medium using a sampling loop. Typically, *V. cholerae* appears as yellow colonies on the TSBC agar, while *V. parahaemolyticus* appears as turquoise. Other colonies (*V. alginolyticus*, *V. metschnikovii*, *V. fluvialis*, *A. hydrophilla* or *A. sobria*) that grew up on the TCBS agar with different morphological features were further identified by the Gram stain and biochemically characterized. All colonies grown on the TCBC agar prior to the identification were subcultured onto a plate of Columbia Blood Agar Base. The identification of the presumptive *Vibrio* species colonies as well as other colonies was performed using the Vitek 2 automated system (BioMérieux, Marcy-l’Étoile, France). 

The identification of *Salmonella* spp. and *Shigella* spp. was performed in accordance with the ISO 19250: 2010. For the identification in seawater, a 1000 mL seawater sample was filtered. For the identification in sediment, a 100 mL of PBS, in which the sediment sample was processed, was filtered. The filtration membrane was transferred to 50 mL of Buffered peptone water (BPW), a *Salmonella* pre-enrichment broth, and incubated at 36 ± 2 °C for 18 ± 2 h. After incubation, the PBS aliquots were transferred to different broths— the Rappaport–Vassiliadis Soya Peptone Broth (RSV Broth) and the Muller–Kauffmann tetrathionate-novobiocin broth (MKTTn)—and incubated at 41.5 ± 1.0 °C for 24 ± 3 h and at 36 ± 2 °C for 24 ± 3 h, respectively [28,29]. For the *Shigella* spp. determination, membranes were incubated in Selenite cystein broth at 37° for 24 h. The cultures obtained in broths were streaked onto a selective Xylose Lysine Deoxycholate (XLD) agar using a sampling loop and incubated at 37 °C for 24 h. The colonies of presumptive *Salmonella* spp. (red colonies with black center) and *Shigella* spp. (red colonies) were confirmed by an analytical profile index (API test, API^®^ ID 32 E, Biomerieux, F) and an agglutination test for *Salmonella* spp.

The results for seawater samples were expressed as CFU/100 mL (CFU—colony forming units). *Salmonella* spp. and *Shigella* spp. were expressed qualitatively (detected/not detected). To enable a comparison of the concentrations in the seawater and sediment, the concentrations of bacteria in the sediment measured as CFU/g were converted to CFU/100 mL, assuming the specific density of water to be approximately 1 g/mL.

### 2.4. Atmospheric and Anthropogenic Factors 

#### 2.4.1. Population

The population estimates were calculated based on the Census of Population, Households and Dwellings 2011, natural change and net migration data by the Croatian Bureau of Statistics. Since the estimates for 2020 were not available, the average of 2017–2019 was used.

#### 2.4.2. Tourists 

The PGKC tourists included the number of tourist nights in Rijeka and Kostrena and in Pula, Fažana and Medulin for the IC tourists, during bathing season. Tourist nights refer to every registered overnight stay of a person (tourist) in an accommodation establishment, including children, regardless of age. Data were retrieved from the Statistical Databases of the Croatian Bureau of Statistics [30], which is the main producer, disseminator and coordinator of the Official Statistical System of the Republic of Croatia, as well as the main representative of the national statistical system in front of the European and international bodies in charge of statistical affairs.

#### 2.4.3. Ballast Water 

Data on BW discharge in the ports of Pula and Rijeka during the 2014–2020 period were retrieved from the Croatian Integrated Maritime Information System (CIMIS) accessed with permission of the Maritime safety directorate of the Ministry of the Sea, Transport and Infrastructure of the Republic of Croatia. BW discharge thresholds are defined by the D-2 standard for toxicogenic *V. cholerae* O1 and O139 (<1 CFU/100 mL or <1 CFU/g of wet weight of a zooplankton sample), *E. coli* (<250 CFU/100 mL) and intestinal enterococci (<100 CFU/100 mL) [31]. 

#### 2.4.4. Precipitation and Solar Radiation

Precipitation data were obtained by the Croatian Meteorological and Hydrological Service (DHMZ, Zagreb) [32], and the solar radiation intensity data were obtained from a satellite database Solcast (retrieved in 2021) [33].

### 2.5. Bathing Water Quality

Bathing water quality was determined in accordance with the criteria set by the EU BWD [2] using the FIB concentration as the basis (Table 1).

### 2.6. Statistical Analysis 

The normality of bacterial count data was assessed with the Shapiro–Wilk’s W-test, which showed that the data did not follow normal distribution for any of parameters measured. All bacterial counts were log10 transformed prior to statistical tests. Spearman’s rank correlation coefficients were calculated to estimate the correlations between different microbial parameters. The microbiological load at different sampling sites was calculated by the Kruskal–Wallis ANOVA and multiple comparisons of mean ranks at the significance level *p* < 0.05. For data originating from routine (official) monitoring of coastal water quality, the 90th and 95th percentile values were calculated in accordance with the procedure described in the EU BWD [2].

### 2.7. Numerical Modeling of the Pollution Source in the Rijeka Area

**Figure 2:**
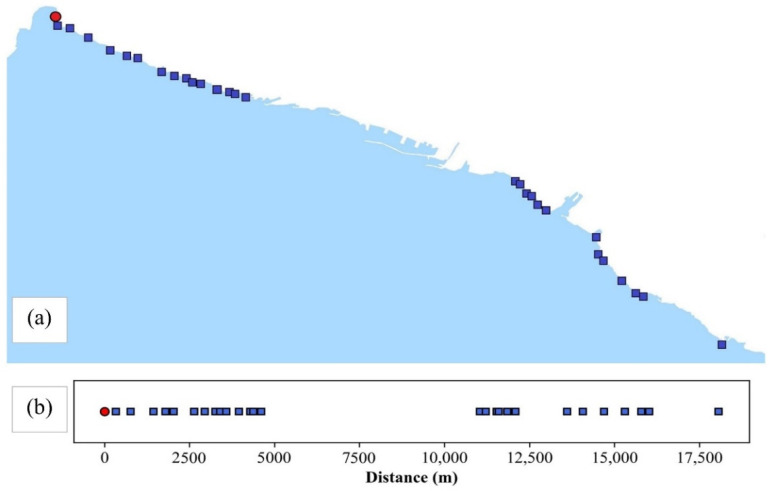
Sampling sites in Rijeka area (**a**). One-dimensional representation of sampling sites along the coast (**b**). The zero reference point is marked red. The entrance to the port of Rijeka is at 7500 m.

The port of Rijeka is situated in the city center. Figure 2a shows the sampling sites positioned on both sides of the port. The 1D representation of the sampling area was used to form the unwound coastal curve with linear distances along the coast, created showing the sampling points’ direct line distances starting from the zero reference sampling point marked red (the most northwestern point, i.e., its distance was set to 0) in Figure 2b, while the other sampling points were shown in blue.

The yearly water quality assessment based on average *E. coli* and intestinal enterococci measured values per sampling site were curve fitted, with the *x*-axis being the distance along the coast from the zero reference point (Figure 2b), while the *y*-axis represents *E. coli* or intestinal enterococci yearly averaged values.

## 3. Results

### 3.1. Microbial Contamination of the Two Ports 

The results of the microbiological parameters analyzed in the ports of Pula and Rijeka are shown in Table 2a,b. The presence of FIB was recorded in all seawater samples at all sites in the autumn and winter and at directly affected BW sites (PUbwC, PUbwS, RIbwB, RIbwS) in the spring and summer. At indirectly affected BW sites (PUchm, RIchm) and reference sites (PUref, RIref), FIB were detected sporadically in seawater samples during the spring and summer. In the sediment samples, FIB were detected in all seasons only at RIbwB and sporadically at other sites. 

The investigation of additional microbiological parameters indicated *C. perfringens* being the most outspread in the seawater and sediment of both ports, especially in the autumn and winter. *P. aeruginosa* was observed in the seawater from the port of Rijeka at BW impacted sites during all seasons. In the seawater samples from other sites in this port and in the sediment in both ports, *P. aeruginosa* was observed only sporadically in the winter and spring. *S. aureus* and *Salmonella* spp. were not detected in the sediment but were observed in seawater samples at RIbwB in almost every season (*S. aureus* absent in the autumn) and sporadically in other seasons and at other sites of the port of Rijeka. *Shigella* spp. and *A. sobria* were not detected, while *A. hydrophilla* was present in the winter seawater samples of BW impacted sites in the port of Rijeka. *V. alginolyticus*, *V. cholerae* non-O1/non-O139, *V. fluvialis*, *V. metschnikovii* and *V. parahaemolyticus* were rarely observed in the sediment and somewhat more frequently in the seawater samples, with the exception of *V. alginolyticus*, which was detected at most sites in the seawater of the port of Pula in the autumn and winter. 

In total, out of 52 port seawater samples, 22 (42.3%) met the D-2 standard. However, in four of them (7.7%), some pathogens were detected. None of the samples were positive for epidemic-causing strains of *V. cholerae*, serogroups O1 and O139. A primarily non-toxic-strain *V. cholerae* non-O1/non-O139 was found in one seawater sample in the port of Rijeka, at RIbwB, in Sep 2014 (*E. coli* and enterococci were 1400 and 600 CFU/100 mL, respectively). 

Among the other pathogenic *Vibrio* species, *V. parahaemolyticus* was found in 10.7% of seawater samples from the port of Pula. In seawater samples from the port of Rijeka, *V. metschnikovii* and *V. fluvialis* were detected in 12.5% and 8.3% of the samples, respectively. *V. alginolyticus* was the most dominant *Vibrio* species in seawater (25.0% and 4.2%) and sediment (21.4% and 13.3%) samples in the ports of Pula and Rijeka, respectively. *Aeromonas* species were found exclusively in seawater samples from the port of Rijeka: *A. hydrophilla* (12.5%) and *A. sobria* (6.6%). In total, *Vibrio* and *Aeromonas* strains were found in 17 out of 52 seawater samples (32.7%), of which 13 (76.5%) did not meet the BW regulatory criteria. Out of 29 sediment samples, 5 (17.2%) contained *V. alginolyticus* and *A. sobria*. A total of 8 out of 20 analyzed seawater samples were positive for *Salmonella* spp. (40%), while in the sediment samples, *Salmonella* spp. was not detected. No *Shigella* was isolated from any seawater or sediment sample.

**Figure 3:**
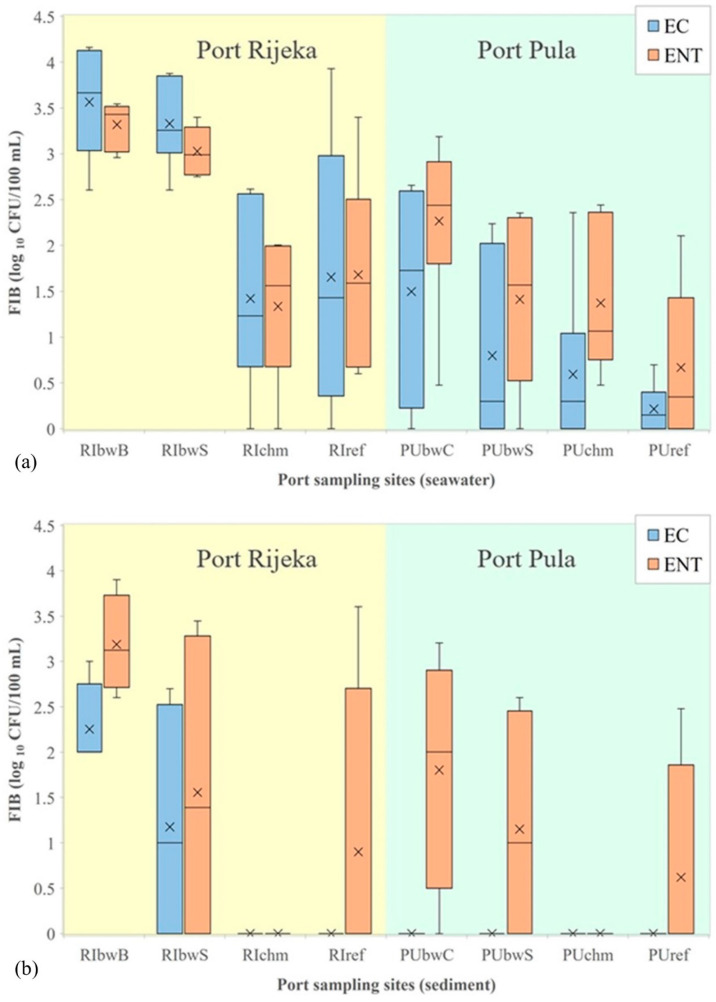
Levels of *E. coli* (EC) and intestinal enterococci (ENT) (logarithmic values) determined in: (**a**) seawater and (**b**) sediment in the ports of Rijeka and Pula. Boxplots show the median (line), average (x), quartiles (boxes) and non-outlier range (whiskers).

In the port of Rijeka, the highest levels of *E. coli*, enterococci and *C. perfringens* in seawater were determined at RIbwB (averages were 6567, 2352 and 359 CFU/100 mL, respectively), whereas at the remaining three sites (RIbwS, RIchm and RIref), the average values were considerably lower (1647, 582 and 59 CFU/100 mL, respectively; Figure 3a, *C. perfringens* not shown). These site-specific differences were statistically significant (Kruskal–Wallis, N = 24, H = 11.806, *p* = 0.008; N = 24, H = 15.492, *p* = 0.001; N = 20, H = 10.336, *p* = 0.001, respectively). Although a higher count of *P. aeruginosa* was also observed in the seawater at RIbwB (average, 50 CFU/100 mL) than at the other sites (average, 19 CFU/100 mL), the difference was not statistically significant (data not shown). *S. aureus* was determined in 6 out of 20 seawater samples, with the maximum abundance of 10 CFU/100 mL measured at RIbwB (data not shown).

No statistical difference was found between the sites in the levels of microbial parameters analyzed in the sediment. In addition, there were no seasonal variations. However, *E. coli* was observed only at RIbwB and RIbwS, and its level was 21 times higher in seawater than in sediment (Mann–Whitney, N = 38; Z = 3.102, *p* = 0.002). In contrast, *C. perfringens* levels in the sediment were 1.5-fold higher than in seawater (Mann–Whitney, N = 31; Z = −4.547, *p* < 0.001). No other statistically significant relations were determined (for intestinal enterococci, *P. aeruginosa* and *S. aureus*). 

In the seawater from the port of Pula, significantly higher concentrations were found only for the intestinal enterococci at PUbwC (average, 493 CFU/100 mL) in comparison with the other three studied sites (averages 0–276 CFU/100 mL; Figure 3a). The observed difference was also statistically significant (Kruskal–Wallis, N = 24, H = 7.112, *p* = 0.048). The intestinal enterococci levels in the winter (average, 406 CFU/100 mL) were significantly higher than in the other seasons (average, 57 CFU/100 mL; Kruskal–Wallis, N = 24, H = 12.102, *p* = 0.017). 

In the port of Pula, higher levels of *E. coli* and intestinal enterococci (average, 2877 and 1024 CFU/100 mL) were detected in seawater than in sediment, where the average level of intestinal enterococci was 186 CFU/100 g, while the level of *E. coli* was below the detection limit (Figure 3b). This difference was statistically significant (Mann–Whitney, N = 38; Z = 4.845, *p* < 0.001; N = 38; Z = 2989, *p* < 0.003). No other statistically significant relations were determined (for *P. aeruginosa* and *S. aureus*). 

Incidentally, in the sediment of both ports, *S. aureus* was not detected (Table 2a).

The comparison of *E. coli* and intestinal enterococci data for seawater indicated a statistically significant difference between the two studied ports (Mann–Whitney, N = 48; Z = 4.178, *p* < 0.001; Z = 2.714, *p* = 0.007, respectively). In the sediment, *E. coli* in the port of Rijeka was present in higher concentration in comparison with the port of Pula (Mann–Whitney, N = 28; Z = 2.663, *p* = 0.008), whereas for intestinal enterococci, the statistical analysis did not reveal differences in concentrations (Figure 3a,b).

### 3.2. Comparison of the Microbial Contamination in Ports with the Surrounding and Wider Areas

The correlation analyses of the complete microbial parameters’ dataset in seawater from the port of Rijeka revealed several positive correlations: *E. coli* and intestinal enterococci/*C. perfringens*, intestinal enterococci and *C. perfringens*/*P. aeruginosa*/*S. aureus*, *C. perfringens* and *P. aeruginosa*. In the sediment, positive correlations were found between *E. coli* and intestinal enterococci/*C. perfringens*. Significant correlations were also revealed between *E. coli* in the sediment and *E. coli*/intestinal enterococci/*C. perfringens*/*S. aureus* in seawater (Table 3a). No correlations were found between any parameters in the port of Pula (Table 3b).

**Figure 4:**
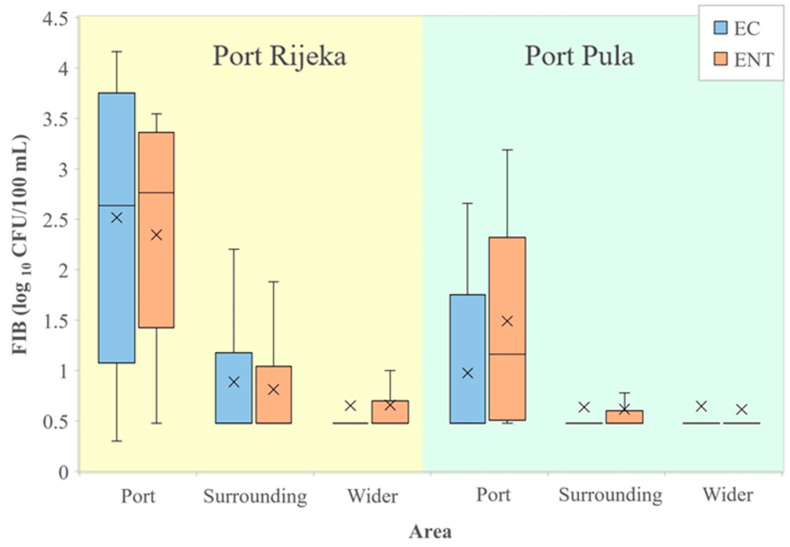
Levels of *E. coli* (EC) and intestinal enterococci (ENT; logarithmic values) determined in the port of Rijeka, the respective surrounding and the wider area (**left**) and in the port of Pula, the respective surrounding and the wider area (**right**) in the period 2014–2015. Boxplots show the median (line), average (x), quartiles (boxes) and non-outlier range (whiskers).

A comparison of FIB values from seawater of the port of Rijeka with the surrounding and wider area (Figure 4, left) showed *E. coli* and intestinal enterococci counts significantly higher in the port than in the surrounding (96.7 and 64.7 times higher, respectively) and wider area (197.4 and 114.1, respectively). In the port, the surrounding and the wider area, the average values of *E. coli* were 2877 vs. 30 vs. 15 and of enterococci 1025 vs. 16 vs. 9 CFU/100 mL, respectively. Likewise, *E. coli* and intestinal enterococci values in seawater from the port of Pula (Figure 4, right) were significantly higher than in the surrounding (5.9 and 19.5 times higher, respectively) and the wider area (6.3 and 23.5, respectively). In the port, the surrounding and the wider area, the average values of *E. coli* were 61 vs. 10 vs. 10 and of intestinal enterococci 174 vs. 9 vs. 7 CFU/100 mL, respectively.

### 3.3. Assessment of the Microbiological Quality of Seawater in the Surrounding and Wider Area

**Figure 5:**
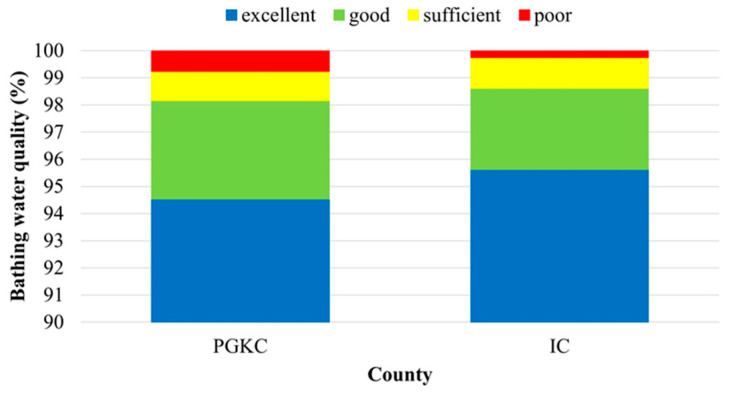
Overall coastal bathing water quality in the Primorje-Gorski Kotar County (PGKC) and Istria County (IC) in the period 2009–2020.

The assessment of a twelve-year seawater quality dataset (2009–2020) obtained from the two counties resulted in a higher average share of bathing sites with excellent quality and a lower share of poor bathing sites in IC (95.6% and 0.2%, respectively) in comparison to PGKC (94.6% and 0.8%, respectively) (Figure 5).

**Figure 6:**
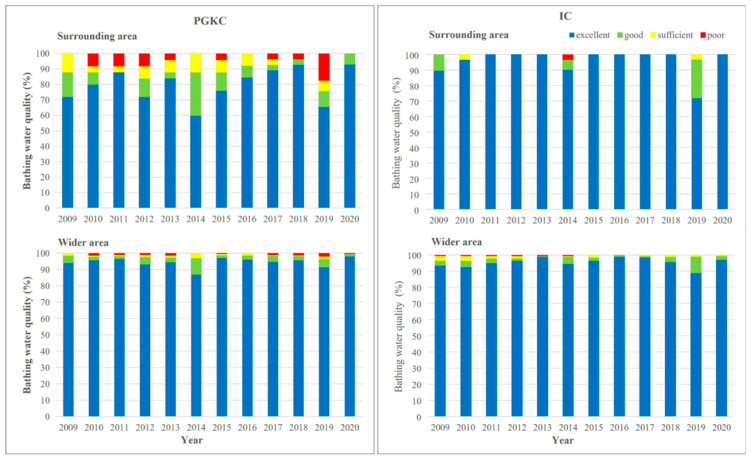
Yearly assessment of bathing water quality in surrounding area (**upper**) and wider area (**lower**) of the port of Rijeka (PGKC; **left**) and the port of Pula (IC; **right**) in the period 2009–2020.

The bathing water quality in the surrounding area of each port and the wider area in the respective county was generally better in the Pula (IC) than in the Rijeka area (PGKC; Figure 6). In PGKC, the water quality in each year of the investigated period was better in the wider than in the surrounding area of the port (Figure 6, left). In contrast, the same analysis of the IC dataset revealed that only in 2009, 2014 and 2019 was the water quality better in the wider area, and that in the other years of the investigated period, the water quality in the surrounding area was better than in the wider area (Figure 6, right).

### 3.4. Atmospheric and Anthropogenic Influences on Seawater Quality in the Area Surrounding Investigated Ports

**Figure 7:**
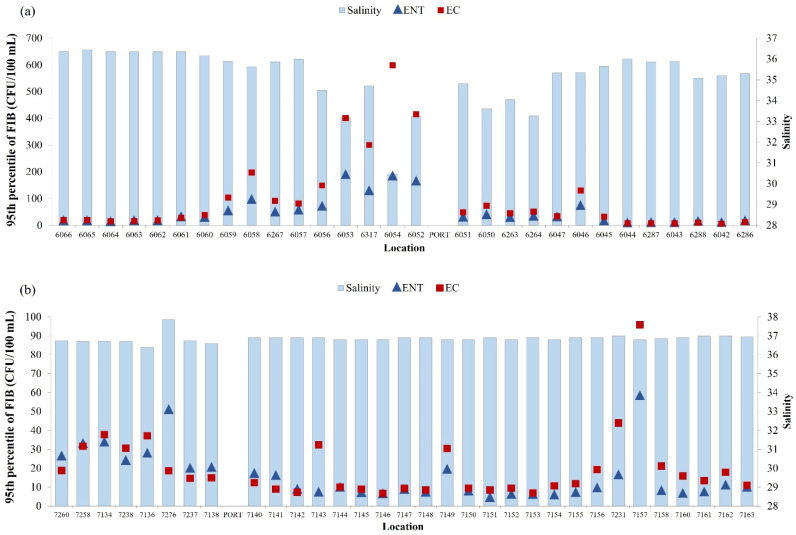
Yearly median of salinity (blue bars) and 95th percentile of *E. coli* (EC—red square) and intestinal enterococci (ENT—blue triangle) in the surrounding area of (**a**) the port of Rijeka and (**b**) the port of Pula.

A negative correlation was found between the level of *E. coli* and intestinal enterococci and salinity of seawater in the surrounding area of the port of Rijeka (Figure 7a; N = 2891, rs = −0.477, *p* < 0.001; N = 2891, rs = −0.456, *p* < 0.001, respectively). The correlations between water temperature and concentration of *E. coli* and intestinal enterococci were also significant but very weak (N = 2891, rs = −0.191, rs = −0.07, *p* < 0.001, respectively).

In the surrounding area of the port of Pula, the correlation analysis gave different results, with positive correlation between *E. coli* and salinity of seawater (N = 3749, rs = 0.232, *p* < 0.001) (Figure 7b). The same positive correlation was determined between the number of *E. coli* and intestinal enterococci and water temperature (N = 3749, rs = 0.195, *p* < 0.001; N = 3749, rs = 0.132, *p* < 0.001, respectively) (data not shown).

**Figure 8:**
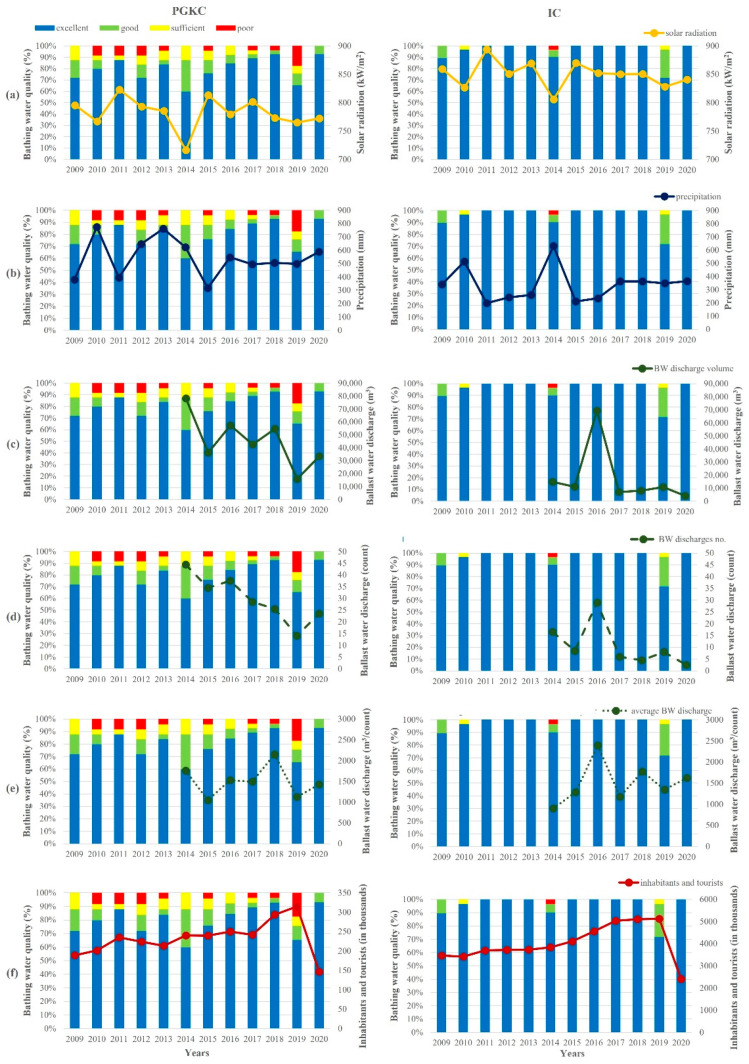
Yearly averages of the bathing water quality in the surrounding area of the port of Rijeka (PGKC; left) and the port of Pula (IC; right) with cumulative values of (**a**) solar radiation, (**b**) precipitation, (**c**) total BW discharge, (**d**) number of BW discharges, (**e**) ratio between total BW discharge and number of discharges and (**f**) number of inhabitants and tourists. Calculations were based on values obtained during bathing seasons (from mid-May to late September) over the 12-year period (2009–2020).

Yearly averages of the bathing water quality in the surrounding area of both ports over a 12-year period (2009–2020) were analyzed against the solar radiation and precipitation intensity, BW discharges and the number of inhabitants with respective tourists during the bathing season (Figure 8). 

The solar radiation in Rijeka was generally lower than in Pula, with a 2009–2020 bathing season average of 782 kW/m^2^ and 850 kW/m^2^, respectively (Figure 8A). The year with the lowest radiation in both regions was 2014, amounting to 716 kW/m^2^ in Rijeka and 806 kW/m^2^ in Pula. In contrast, precipitation (Figure 8B) in Rijeka was generally more abundant than in Pula, ranging from 316 mm in 2015 to 775 mm in 2010, with a period average of 544 mm in Rijeka and from 199 mm in 2011 to 632 mm in 2014 with a period average of 339 mm in Pula. In Rijeka, 2009, 2011 and 2015 were the years with low amounts of precipitation, whereas 2010 and 2013 were ones marked with especially abundant precipitation. In Pula, precipitation was more often under the period average (in years 2011, 2012, 2013, 2015 and 2016), while it was higher only in 2010 and 2014.

The data on BW discharge in the two ports were only available for the period 2014–2020. In that period, the total discharged amount of BW (Figure 8C) and the number of BW discharges (Figure 8D) had a decreasing trend in the port of Rijeka, from 78,198 m^3^ to 33,558 m^3^ and 45 to 24 discharges, respectively, with the lowest amount of 15,733 m^3^ and 14 discharges in 2019. In Pula, a similar situation was observed—a decrease in the total discharged amount of BW from 14,824 m^3^ to 4050 m^3^ and from 17 to 3 discharges. In 2016, a distinctly higher discharge of 69,397 m^3^ in 29 separate discharges occurred. On average, in the port of Rijeka, an average of 30 BW discharges occurred, each amounting to an average of 45,489 m^3^, whereas in the port of Pula, the averages amounted to 11 discharges and 17,861 m^3^. However, the ratio of discharged BW per number of discharges in both ports was similar and amounted to an average of around 1500 m^3^ per single discharge in the investigated period (Figure 8E).

The number of inhabitants and tourists in the surrounding area of the port of Rijeka increased from 188,879 in 2009 to an average of 234,788 in the period 2011–2017, followed by an increase to 293,576 in 2018 and 314,625 in 2019 (Figure 8F). A considerable decrease marked the year 2020, with only 146,597 tourists in this region, related to the circumstances of the COVID-19 pandemic. In the surrounding area of the port of Pula, there was also a steady increase from 3,469,147 in 2009 up to 4,104,883 in 2015, followed by a more distinct increase to 4,559,346 in 2016 and to an average of 5,090,305 in the period 2017–2019. In 2020, there followed a marked decrease to 2,402,412.

### 3.5. Numerical Modeling of the Pollution Source in the Rijeka Area

**Figure 9:**
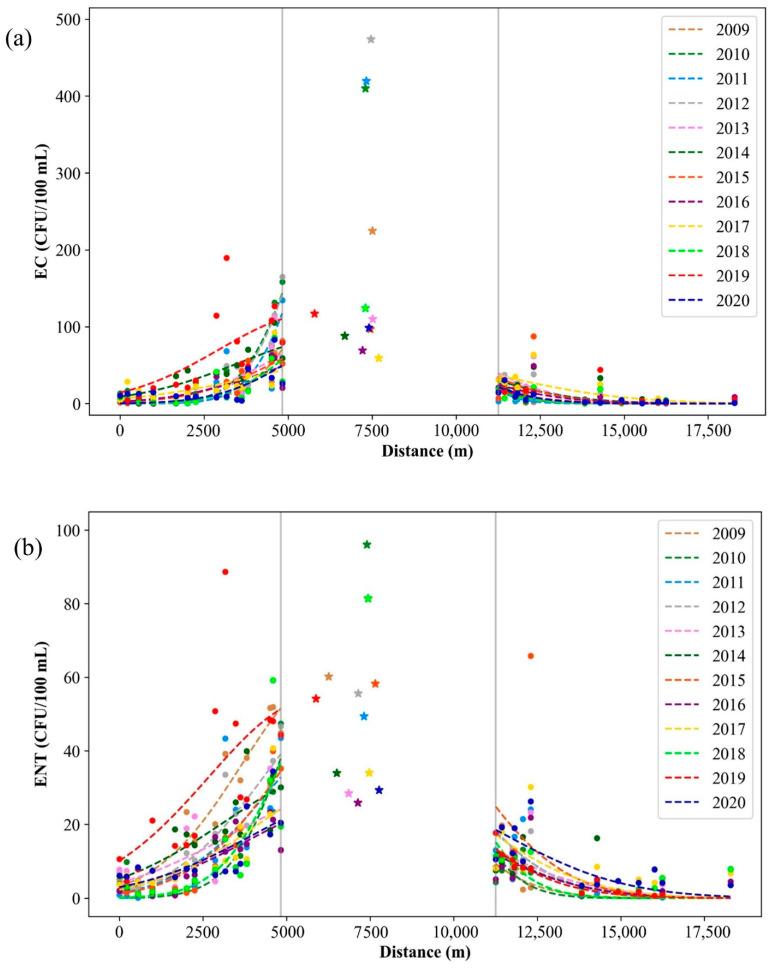
Yearly averaged (**a**) *E. coli* (EC) and (**b**) intestinal enterococci (ENT) in the port of Rijeka (stars) and the surrounding area of the port (dots) during the period of 2009–2020.

**Figure 10:**
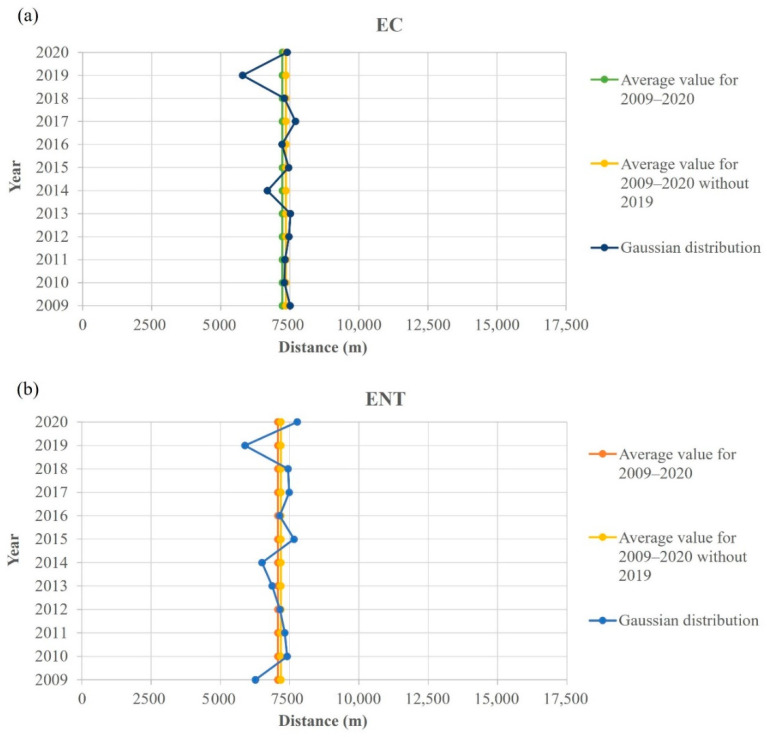
Pollution dispersion Gaussian function maximum point spatial shift regarding the 12-year average for (**a**) *E. coli* (EC) and (**b**) intestinal enterococci (ENT) from the entrance to the port of Rijeka (7500 m).

An interesting spatial behavior was noticed when the Gauss fitted function maxima, representing the pollution source location of *E. coli* and intestinal enterococci, was calculated (Figure 9), where two vertical gray lines at distances 4.826 m and 11.262 m represent the edges of the port of Rijeka. As visible in Figure 10, which shows the yearly *E. coli* and intestinal enterococci Gauss function maxima position covering the whole 12-year period, the maximum point is placed in the port of Rijeka, except for the year 2019, when the maximum point shifts significantly leftward, i.e., indicating the spatial shift of the dominant pollution source area.

## 4. Discussion

We studied the microbial contamination of seawater and sediments in two ports in the northeastern Adriatic Sea, the impact of the ports on the surrounding coastal bathing waters and the potential impact of ballast water transfer to other areas. The long-term microbiological quality of seawater in the coastal area surrounding the ports and in the wider coastal area was also assessed, and the possible atmospheric and anthropological influences on the studied areas were examined.

Our investigation indicates that acceptable abundances of *E. coli* and intestinal enterococci, as defined by the D-2 standard, do not indicate a complete absence of pathogen-induced risks, as, occasionally, additional indicators and pathogens, such as *C. perfringens*, *S. aureus*, *P. aeruginosa*, *Salmonella* spp., *A. hydrophilla*, *A. sobria* and *Vibrio* spp., were detected in the D-2 standard compliant samples. The only investigated pathogen that remained undetected in our research was *Shigella* spp.

The larger and continuous FIB load in the port of Rijeka recorded in the study might be attributed to two main sources. First, as a large urban center, the higher number of inhabitants probably contributed to a higher microbiological load in the coastal water. This area was already reported as susceptible to fecal pollution, either by uncontrolled sewage outfalls or by groundwater bringing fecal contamination [34,35]. Second, as the largest port on the Croatian coast, this port has significantly higher traffic intensity and BW discharge, both potential sources of microbial pollution. The absence of any seasonality of pollution in the port of Rijeka suggests a continuous pressure from microbiological pollution in this area, either from land-based and/or BW discharge. Another contributing factor seems to be the port’s configuration. The port of Rijeka consists of two enclosed basins. Each basin only has one opening to the open sea, at the western side of the basin, which might induce the retention of microbiological load and limit its spread into the open sea. This also provides an explanation for the two most polluted sites in the study, located in these two basins. On the other hand, in the port of Pula, a higher prevalence of enterococci in seawater and the complete absence of *E. coli* in the sediment indicate sporadic pollution, while in the port of Rijeka, pollution is reported more frequently. This is consistent with the finding of a slower die-off rate of enterococci than *E. coli* in saline water [36]. The higher concentrations of enterococci, but not *E. coli*, in the winter could be due to sporadic and lower levels of contamination, so *E. coli* was not recorded because of the low frequency of the sampling and its faster die-off rate.

The positive correlation between *E. coli*, enterococci and *C. perfringens* in the seawater and the sediment of the port of Rijeka indicates their concomitant and continuous inflow. The results indicate better survival and longer retention of *C. perfringens* than FIB in sediment, given their recorded seawater/sediment ratios. This could be a result of longer persistence of *C. perfringens* spores than coliforms or streptococci, as suggested by Skanavis and Yanko [37], making *C. perfringens* a better indicator of area contamination. Although concomitantly present in seawater during all seasons, *P. aeruginosa* was scarcely determined in sediment and only in the winter and spring, thus indicating better resilience at lower temperatures, as established by Burkhardt et al. [38]. The absence of any correlation between microbial parameters in the port of Pula could be explained by sporadic contamination and different die-off rates of the studied bacteria. 

The average bathing water quality was generally better throughout PGKC than at the sites in the surrounding area of the port of Rijeka, indicating potential impact of the port. Particularly in 2009–2015 and 2019, microbiological pollution from the port may have affected water quality in the surrounding area, as sites rated below average quality are located in close proximity to the port. During the summer period, a westward circulation pattern along the northern coast of the Rijeka Bay where the port of Rijeka is located, along with the predominant impact of easterly winds, facilitates the waters to leave both basins of the port of Rijeka, as the openings of both basins are at their west side [39]. The circulation along the northern and western coast of the Rijeka Bay is cyclonic (counterclockwise) and therefore favors water circulation along the coast, where the bathing sites are located, toward the open sea, where waters ultimately enter the open cyclonic circulation of the Adriatic Sea. On the other hand, in 2016–2018 and 2020, the sites of under-excellent bathing water quality were located too far from the port to become affected directly by its microbiological load and, incidentally, in these years, the bathing water quality in the surrounding area was generally better than in the aforementioned years.

In contrast, only in 2019 could microbiological load from the port of Pula have affected the surrounding area of the port, when the impacted bathing sites were in close proximity to the entrance of the Bay (not shown). This assumption corroborates the cyclonic circulation pattern along the Adriatic coast, passing northward along the entrance to the port of Pula and the predominant easterly winds facilitating waters to leave the port (sensu the Pula Bay) [39]. It is also in line with the fact that in 2018 and 2019, the number of tourists was rather similar, excluding this factor from imposing any effect. In other years, the sites rated good, sufficient or poor in the surrounding area of the port of Pula were located too far from the respective port to receive its influence. Furthermore, in the Pula region generally, bathing water quality was better in the surrounding area compared to the complete dataset of the IC, which is in line with our finding that the microbiological load from the port rarely affects the surrounding area. We presume that the open basin of the Pula Bay favors the immediate spreading of any load imposed from the ships’ BW and its dilution in the Bay. However, this further implies that some other factors impact the bathing quality in this County. 

In the surrounding area of the port of Rijeka, in contrast to the port of Pula, a negative correlation was found between FIB concentration and salinity, as well as seawater temperature. This is particularly pronounced at sites 6054 and slightly less so at 6052 and 6053, which is related to the presence of a greater number of coastal springs. Freshwater inflow from the land, most likely from the municipal sewage and post-rainfall runoff, is often microbiologically contaminated and has a lower temperature than the coastal sea [40,41]. The exception is one location in IC, which showed the highest concentration of fecal indicators but with average salinity values (average, 36.3). This beach (Sandy Bay, Medulin) was classified as poor in 2014, resulting from the water overflow from the wastewater drainage system to the beach due to floods caused by extremely heavy rains.

Solar radiation and precipitation are two of the most influential parameters regarding the microbiological coastal water quality [9]. Our results support this finding. The lower bathing water quality in the surrounding area of the port of Pula in 2010, 2014 and 2019 coincided with a lower solar radiation and higher precipitation. Despite the high number of tourists in IC from 2017 to 2019, bathing water quality in 2017 and 2018 was excellent, presumably due to sufficient radiation and moderate precipitation. However, the lower radiation intensity in 2019, as the main factor affecting the die-off rate of FIB [42,43,44,45], seemed to add to the tourist impact and resulted in the worst bathing water quality over the entire research period.

The importance of atmospheric conditions for bathing water quality was further supported by the results in the Rijeka area, where the bathing waters were generally of lower quality, as radiation was lower and precipitation higher than in the Pula area. However, the outcome on the quality of bathing water is not as straightforward. In 2018, moderate radiation, precipitation and BW discharge with a high number of tourists resulted in one of best rated bathing waters of the investigated period. Nevertheless, in 2017, a more intense radiation with similar precipitation, lower BW discharge and considerably lower number of tourists than in 2018 resulted in a slightly lower quality of bathing water than in 2018. However, all the distinguished factors, anthropogenic (BW discharge and population with tourists) and atmospheric (solar radiation and precipitation), especially when combined in the same direction regarding the quality of bathing water, contributed to the outcome. This is shown by the 2014 and 2019 cases in Rijeka, where the low radiation intensity and high BW discharge in 2014 and the record-breaking tourism year in 2019 were most likely the causes of the poorer bathing water quality in the studied period.

To identify more clearly the influence of tourists on the bathing water quality, we focused on two outstanding years, 2019 and 2020. We contrasted 2019, a record tourist year, with 2020, which was a specific COVID 19 pandemic-affected year. The drop in tourist numbers from 2019 to 2020 was 53%. Strangely enough, the decrease was exactly the same in Rijeka and Pula. In the same period, the average increase in water quality rated as excellent in the areas of Rijeka and Pula was 40%. Two years in a row resulted in a substantially different value of tourist numbers because of the COVID-19 pandemic. Therefore, this comparison is a unique opportunity to determine the impact of the tourism industry on bathing water quality and the global environment. The decrease in the number of tourists in Rijeka was high but not as high as the extreme number in Pula. Nevertheless, the water quality in Rijeka decreased correspondingly even more, which leaves room to look for other negative environmental impacts in this area. To account for additional possible influences, we assessed the area with a modeling approach. In all the investigated years (2009–2020), the pollution source was clearly in the port. Only in 2019 was it dislocated westward. We believe a possible explanation might be for the following reason. During the period of late 2018 and during 2019, in the northwestern end of the port of Rijeka, the biggest Croatian container terminal was built. These activities caused long-term turbidity, which blocked solar irradiation and resulted in increased microbiological pollution. 

FIB and the pathogens that are harbored in marine sediments [46] may be resuspended into water naturally by tides, waves, storm water runoff or by anthropogenic activities: recreational, bottom dredging, trawling, shipping traffic, BW exchange. These sediment disturbances can degrade the seawater quality and pose a risk for the spread of pathogens [11,47]. Numerous studies demonstrated higher concentrations and longer persistence of indicator bacteria and pathogens adsorbed to the sediment relative to the overlying seawater [40,48,49,50,51]. 

In the present study, *C. perfringens* was frequently observed in seawater; however, it was also the only pathogen observed more often and in higher abundances in the sediment than in the seawater. This is consistent with the findings of Skanavis and Yanko [37] that the spore-forming obligate anaerobe *C. perfringens* is more persistent in sediment than *E. coli* or enterococci. As in the present study, Chiaretti et al. [18] observed the highest load of *C. perfringens* in the sediment of large ports, which are characterized by favorable conditions for longer survival of these bacteria, such as fine-grained sediment with higher organic matter content [5]. Because of its properties, *C. perfringens* is considered a persistent indicator of a long-term fecal contamination [4] and the presence of protozoan cysts end enteric viruses [52]. This is in line with the findings in the port of Rijeka where FIB and *C. perfringens* were mostly detected concurrently in seawater samples. It is routinely used in the U.S. state of Hawaii as an additional indicator of fecal contamination associated with the proliferation of *E. coli* and enterococci in tropical and subtropical waters and sediments [53]. 

The beach sand and sediment are not examined in the routine monitoring of beaches, although numerous studies have pointed to beaches as a diffuse source of contamination [6,12,54]. Recent WHO guidelines on recreational water quality recommended the inclusion of intestinal enterococci in beach sand monitoring [14]. *C. perfringens* was commonly observed with FIB in the seawater and in the sediment in this study; therefore, we recommend the future use of *C. perfringens* as an indicator for monitoring beach sand over FIB to improve the insight into bathing water quality when paired with FIB in seawater.

## 5. Conclusions

Generally, the concentration of the studied bacteria was higher in the seawater than in the corresponding sediment, except for *C. perfringens*. *C. perfringens* was more frequent in the sediment than FIB and any other pathogen. Accordingly, the monitoring of *C. perfringens* in beach sand might be an excellent addition to the monitoring efforts of seawater quality during the bathing season.

The investigated ship ports, especially the port of Rijeka, were proven to be microbiologically loaded areas, which should be monitored for presence of a wide range of pathogens. As such, ports pose risks to the surrounding areas, often used for recreational purposes. Additionally, they act as potential donors of microbiologically loaded ballast water for other ports.

In addition to solar radiation and precipitation, human activity generated by tourists and locals proved to have significant effects on the bathing water quality. However, our research also indicated the complexity of their influences and that location-specific behavior has to be accounted for, which demands further investigation to be fully understood.

## Figures and Tables

**Table 1:** EU standards for assessment of coastal and transitional waters’ quality at the end of bathing season and for three preceding bathing seasons.

Parameters	Excellent	Good	Sufficient
Intestinal enterococci (CFU/100 mL)	≤100 *	≤200 *	≤185 **
*E. coli* (CFU/100 mL)	≤250 *	≤500 *	≤500 **

* Based upon a 95-percentile evaluation; ** Based upon a 90-percentile evaluation.

**Table 2:** Seasonal presentation of microbial contamination in the ports of Pula and Rijeka.

(**a**) Seasonal presentation of *E. coli* (EC), intestinal enterococci (ENT), *C. perfringens* (CP), *P. aeruginosa* (PA), *S. aureus* (SA) determined in seawater (sw) and sediment (sed) in the ports of Pula (PUbwC, PUbwS, PUchm and PUref) and Rijeka (RIbwB, RIbwS, RIref and RIchm). Abundance in CFU/100 mL; information not available/analysis not processed (n.a.).																			
	**Bacteria**	**EC**				**ENT**				**CP**				**PA**				**SA**	
**Season**	**Station**	**sw**		**sed**		**sw**		**sed**		**sw**		**sed**		**sw**		**sed**		**sw**	**sed**
autumn	PUbwC	53		0		282		100		n.a.		1400		n.a.		0		n.a.	0
	PUbwS	44		0		54		0		n.a.		1600		n.a.		0		n.a.	0
	PUchm	2		0		11		0		n.a.		2100		n.a.		0		n.a.	0
	PUref	1		0		2		0		n.a.		4000		n.a.		0		n.a.	0
	RIbwB	950		100		1005		1100		85		3200		27		0		0	0
	RIbwS	1600		0		775		0		130		1400		25		0		0	0
	RIchm	19		0		35		0		5		3400		0		0		2	0
	RIref	4286		0		1252		0		1		4800		0		0		0	0
winter	PUbwC	413		0		1100		100		n.a.		13,600		n.a.		0		n.a.	0
	PUbwS	86		0		209		400		n.a.		2000		n.a.		200		n.a.	0
	PUchm	114		0		246		0		n.a.		39,000		n.a.		0		n.a.	0
	PUref	2		0		71		0		n.a.		17,200		n.a.		0		n.a.	0
	RIbwB	13,750		100		2900		400		545		22,000		90		100		5	0
	RIbwS	4650		500		1180		2800		274		31,600		105		0		0	0
	RIchm	380		0		99		0		15		0		6		0		0	0
	RIref	235		0		87		0		32		14,400		1		0		0	0
spring	PUbwC	1		0		192		0		n.a.		31,200		n.a.		0		n.a.	0
	PUbwS	3		0		0		0		n.a.		20,800		n.a.		0		n.a.	0
	PUchm	0		n.a.		6		n.a.		n.a.		n.a.		n.a.		n.a.		n.a.	n.a.
	PUref	0		0		0		0		n.a.		5200		n.a.		0		n.a.	0
	RIbwB	6900		1000		2800		8000		100		31,200		3		400		4	0
	RIbwS	6900		0		2500		600		0		20,800		4		200		1	0
	RIchm	7		n.a.		20		n.a.		8		n.a.		2		n.a.		0	n.a.
	RIref	0		0		3		0		3		1800		4		0		0	0
summer	PUbwC	0		0		2		1600		n.a.		23,000		n.a.		0		n.a.	0
	PUbwS	0		0		12		100		n.a.		3600		n.a.		0		n.a.	0
	PUchm	3		n.a.		2		n.a.		n.a.		n.a.		n.a.		n.a.		n.a.	n.a.
	PUref	1		0		0		300		n.a.		11,000		n.a.		0		n.a.	0
	RIbwB	3100		100		3500		1600		520		5200		40		0		2	0
	RIbwS	400		100		1000		0		100		18,400		26		0		0	0
	RIchm	0		0		0		0		0		20,800		0		0		0	0
	RIref	2		0		100		4000		0		18,000		6		0		1	0
(**b**) Seasonal presentation of *Salmonella* spp. (SM), *Shigella* spp. (SH), *A. hydrophilla* (AH), *A. sobria* (AS), *V. alginolyticus* (VA), *V. cholerae* non-O1/non-O139 (VCn), *V. fluvialis* (VF), *V. metschnikovii* (VM) and *V. parahaemolyticus* (VP) determined in seawater (sw) and sediment (sed) in the ports of Pula (PUbwC, PUbwS, PUchm and PUref) and Rijeka (RIbwB, RIbwS, RIref and RIchm). Presence (•), absence (0), inconclusive results (+), information not available/analysis not processed (n.a.).																			
	**Bacteria**	**SM**		**SH**		**AH**		**AS**		**VA**		**VCn**		**VF**		**VM**		**VP**	
**Season**	**Station**	**sw**	**sed**	**sw**	**sed**	**sw**	**sed**	**sw**	**sed**	**sw**	**sed**	**sw**	**sed**	**sw**	**sed**	**sw**	**sed**	**sw**	**sed**
autumn	PUbwC	n.a.	0	n.a.	0	0	0	0	0	0	0	0	0	0	0	0	0	0	0
	PUbwS	n.a.	0	n.a.	0	0	0	0	0	0	0	0	0	0	0	0	0	0	0
	PUchm	n.a.	0	n.a.	0	0	0	0	0	0	0	0	0	0	0	0	0	0	0
	PUref	n.a.	0	n.a.	0	0	0	0	0	0	0	0	0	0	0	0	0	0	0
	RIbwB	•	0	0	0	0	0	0	0	0	0	0	0	0	0	0	0	0	0
	RIbwS	0	0	0	0	0	0	0	0	0	0	•	0	0	0	0	0	0	0
	RIchm	0	0	0	0	0	0	0	0	0	0	0	0	•	0	0	0	0	0
	RIref	0	0	0	0	0	0	0	0	•	0	0	0	0	0	0	0	0	0
winter	PUbwC	n.a.	0	n.a.	0	0	0	0	0	•	0	0	0	0	0	0	0	0	0
	PUbwS	n.a.	0	n.a.	0	0	0	0	0	•	+	0	0	0	0	0	+	0	0
	PUchm	n.a.	0	n.a.	0	0	0	0	0	0	0	0	0	0	0	0	0	0	0
	PUref	n.a.	0	n.a.	0	0	0	0	0	•	0	0	0	0	0	0	0	0	0
	RIbwB	•	0	0	0	•	0	0	0	0	0	0	0	0	0	•	0	0	0
	RIbwS	0	0	0	0	•	0	0	0	0	0	0	0	0	0	0	0	0	0
	RIchm	•	0	0	0	•	0	0	0	0	0	0	0	0	0	0	0	0	0
	RIref	•	0	0	0	0	0	0	0	0	0	0	0	0	0	0	0	0	0
spring	PUbwC	n.a.	0	n.a.	0	0	0	0	0	0	0	0	0	0	0	0	0	0	0
	PUbwS	n.a.	0	n.a.	0	0	0	0	0	0	+	0	+	0	0	0	0	0	0
	PUchm	n.a.	n.a.	n.a.	n.a.	0	n.a.	0	n.a.	0	n.a.	0	n.a.	0	n.a.	0	n.a.	0	n.a.
	PUref	n.a.	0	n.a.	0	0	0	0	0	0	+	0	+	0	0	0	0	0	0
	RIbwB	•	0	0	0	0	0	0	0	0	0	0	0	0	0	•	0	0	0
	RIbwS	0	0	0	0	0	0	0	0	0	+	0	+	0	0	•	0	0	0
	RIchm	0	n.a.	0	n.a.	0	n.a.	0	n.a.	0	n.a.	0	n.a.	0	n.a.	0	n.a.	0	n.a.
	RIref	0	0	0	0	0	0	0	0	0	0	0	0	0	0	0	0	0	0
summer	PUbwC	n.a.	0	n.a.	0	0	0	0	0	0	0	0	0	0	0	0	0	0	0
	PUbwS	n.a.	0	n.a.	0	0	0	0	0	0	0	0	0	0	0	0	0	0	0
	PUchm	n.a.	n.a.	n.a.	n.a.	0	n.a.	0	n.a.	0	n.a.	0	n.a.	0	n.a.	0	n.a.	0	n.a.
	PUref	n.a.	0	n.a.	0	0	0	0	0	0	0	0	0	0	0	0	0	0	0
	RIbwB	•	0	0	0	0	0	0	0	0	0	0	0	0	0	0	0	0	0
	RIbwS	0	0	0	0	0	0	0	0	0	•	0	0	+	0	+	0	0	0
	RIchm	0	0	0	0	0	0	•	0	0	0	0	0	0	0	0	0	0	0
	RIref	0	0	0	0	0	0	0	0	0	0	0	0	0	0	0	0	0	0

**Table 3:** Correlation coefficients among the complete microbial parameters’ dataset in seawater and sediment of the port of Rijeka (**a**) and the port of Pula (**b**). Statistically significant correlations marked in bold.

**a**										
**PARAMETERS**		**Water**					**Sediment**			
		***E. coli***	**Int. enterococci**	***C. perfrigens***	***P. aeruginosa***	***S. aureus***	***E. coli***	**Int. enterococci**	***C. perfrigens***	***P. aeruginosa***
Water	*E. coli*	1.000								
	Int. enterococci	**0.886**	1.000							
	*C. perfrigens*	**0.651**	**0.690**	1.000						
	*P. aeruginosa*	0.459	**0.594**	**0.584**	1.000					
	*S. aureus*	0.530	**0.571**	0.517	0.017	1.000				
Sediment	*E. coli*	**0.588**	**0.625**	**0.625**	0.550	**0.587**	1.000			
	Int. enterococci	0.469	0.517	0.121	0.388	0.434	**0.602**	1.000		
	*C. perfrigens*	0.404	0.367	−0.002	0.129	0.306	**0.566**	0.511	1.000	
	*P. aeruginosa*	0.507	0.462	−0.096	−0.184	0.256	0.232	0.441	0.439	1.000
**b**										
**PARAMETERS**		**Water**					**Sediment**			
		***E. coli***	**Int. enterococci**				***E. coli***	**Int. enterococci**	***C. perfrigens***	***P. aeruginosa***
Water	*E. coli*	1.000								
	Int. enterococci	0.096	1.000							
Sediment	*E. coli*	-	-				1.000			
	Int. enterococci	−0.152	0.093				-	1.000		
	*C. perfrigens*	−0.329	−0.029				-	−0.162	1.000	
	*P. aeruginosa*	−0.288	0.380				-	0.421	−0.310	1.000

## Data Availability

The data presented in this study are available on request from the corresponding author.
